# BIM-integrated carbon footprint assessment for sustainable buildings using real-time monitoring and optimization

**DOI:** 10.1038/s41598-026-47914-8

**Published:** 2026-05-04

**Authors:** Sixu Yu, Yuyang Zhang

**Affiliations:** 1 School of Media and Communication, Shanghai Jiao Tong University, Shanghai, China; 2 Department of Civil Engineering, University of Nottingham, Nottingham, UK

**Keywords:** Building information modeling, Internet of Things, Carbon footprint assessment, Real-time optimization, LSTM prediction, Energy science and technology, Engineering, Environmental sciences, Mathematics and computing

## Abstract

The substantial contribution of the building sector to global carbon emissions highlights the limitations of conventional static assessment approaches and motivates the exploration of real-time, operation-oriented carbon management strategies. This study proposes and evaluates an integrated Building Information Modeling (BIM)–Internet of Things (IoT)–Artificial Intelligence (AI) framework for dynamic carbon footprint monitoring and operational optimization in green buildings. The proposed framework focuses on system-level integration and closed-loop decision support, rather than on the development of novel AI algorithms. A four-layer architecture is designed to integrate BIM-based static building information with IoT-driven real-time operational data, enabling continuous carbon assessment and multi-objective operational optimization. The framework was deployed over a 12-month period in a 15,000 m^2^ LEED Gold-certified office building. Under the specific climatic, operational, and occupancy conditions of the case study, a 26.5% reduction in operational carbon emissions was observed relative to a baseline of 10,000 tCO_2_e, while maintaining acceptable occupant comfort levels. The LSTM-based forecasting module achieved a 24-h prediction accuracy of approximately 92%, supporting short-term proactive operational adjustments. Continuous monitoring further indicated that approximately 18% of operational emissions occurred during unoccupied periods, a pattern that would not be identifiable through static assessment methods. A techno-economic evaluation suggests that the proposed framework is financially feasible within the examined context, with an estimated simple payback period of 1.7 years and an internal rate of return of approximately 42%.

## Introduction

**Fig. 1:**
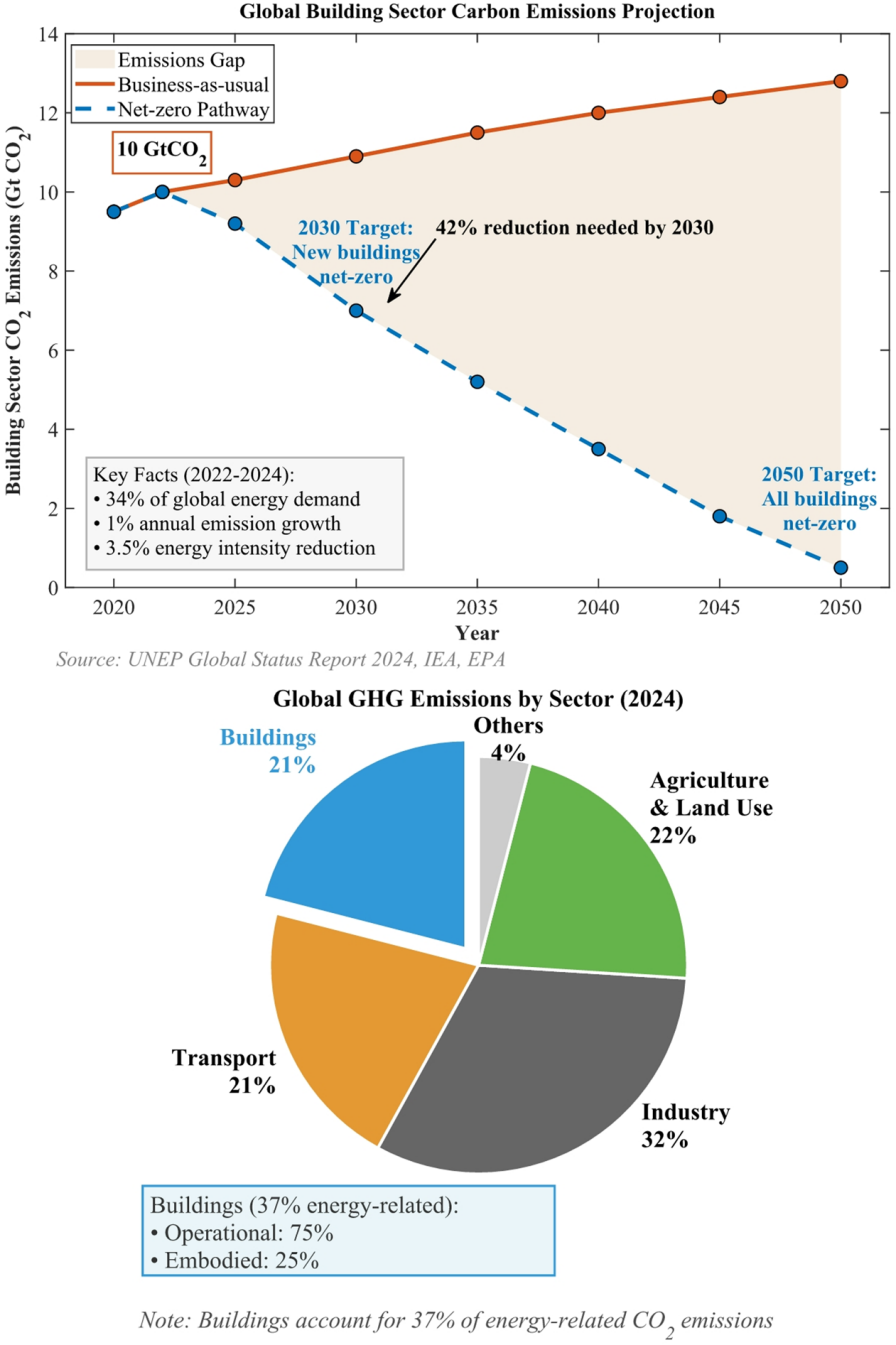
Building sector carbon emissions: current status and future pathways.

The construction industry is one of the leading causes of global climate change and in urgent need of rapid, revolutionary transformation. As illustrated in Fig. 1, buildings currently produce 21% of global warming gas emissions and 37% of energy-related CO_2_ emissions, representing 75% of these emissions as operational emissions, with embodied carbon representing 25% of the sector as a whole^1^. Emerging trends are that unless radical action is taken, building sector emissions continue to increase from 10 GtCO_2_ in 2022 to over 12.8 GtCO_2_ by 2050, whereas to meet the Paris Agreement goals there needs to be a 42% reduction by 2030^2^. The widening gap between trends and climate objectives therefore indicates the need for rapid, revolutionary change for carbon management in the built environment.

The building sector has, hitherto, had traditional assessment schemes that mainly involve static, once-off measurements at design or at the post-construction stage^3^. As long as such practices ensure vital baseline data, such measures do not capture, effectively, the time-varying building activities as well as time-varying energy utilization patterns^4^. The sector has registered 1% yearly growth of emissions as well as yearly energy intensity improvement of 3.5%, which confirms that over-dependence on energy improvement efficiency is inefficient to attain the desired rate of decarbonization^5^. Such paradox justifies the inefficacy of traditional evaluation models, which do not offer real-time responses on time for timely adjustment measures^6^.

The most recent technology innovations of Building Information Modeling (BIM) have redefined building industries’ sustainability assessment^7^. It allows accurate material quantification as well as lifecycle assessment, thereby offering a virtual basis of carbon footprint calculation^8^. Thus, however, current building information modeling practices continue to remain design- as well as, primarily, construction-centered, although sparingly incorporated into operational building stewardship^9^. Likewise, although successfully there has remained implementation of Life Cycle Assessment (LCA) methodology on building information modeling platforms, such implementations continue to remain primarily static as well as historical^10^. The Internet of Things (IoT) technologies development embodies unprecedented environmental monitoring possibilities, real-time, whose integration, however, into dynamic carbon stewardship through building information modeling has yet to materialize^11^.

Recent studies exposed some inherent deficiencies of existing carbon evaluation practice. First, interoperability deficiency of operational data and static BIM models hinders precise monitoring of carbon footprints at the building use phase^12^. Secondly, no predictive functions of existing schemes exist that would support proactive carbon control measures^13^. Without inherent optimization functions, response activities upon detection of inefficiencies are subject to human control, offering slow suboptimal performance^14^. Such vulnerabilities as a system deter the building sector from experiencing fast decarbonization despite the availability of state-of-the-art digital technologies^15^.

This research bridges such gaps through the development of the integrated framework of BIM-IoT-AI for dynamic carbon footprint computation and carbon optimization of green buildings. Based on the extensive building data of BIM, real-time sensing of IoT, and prediction, optimization, and learning capabilities of AI, the system constitutes a dynamic carbon management system^16^. Unlike the traditional approach to dealing with such technologies in isolation, the system provides free data interoperation among static building data as well as real-time operating values, enabling continuous monitoring of carbon footprint as well as automated optimization mitigation^15^.

This work strengthens real-time operational carbon management by moving beyond static assessment toward a monitoring–prediction–optimization workflow. The main contributions are:

1. A BIM-enabled semantic mapping and data fusion pipeline that links IoT telemetry to BIM objects and operational zones, enabling traceable, real-time carbon accounting at the equipment and zone levels.
2. A dynamic carbon footprint assessment method that captures time-varying operational inefficiencies; in the case study, continuous monitoring revealed that ~ 18% of operational emissions occurred during unoccupied periods, which would be obscured by static assessment.
3. A forecasting-informed operational optimization loop that integrates 24-h LSTM prediction with control decision-making under occupant comfort constraints, achieving a 26.5% total emission reduction relative to a 10,000 tCO_2_e baseline.
4. A techno-economic evaluation demonstrating practical feasibility (simple payback: 1.7 years; IRR: ~ 42%), supporting adoption beyond proof-of-concept.

Unlike existing studies that primarily focus on either static BIM-based assessment or isolated IoT monitoring, this study proposes a fully integrated BIM–IoT–AI framework that enables continuous, real-time carbon assessment and closed-loop operational optimization at the system level.

## Methodology

### System architecture

**Fig. 2:**
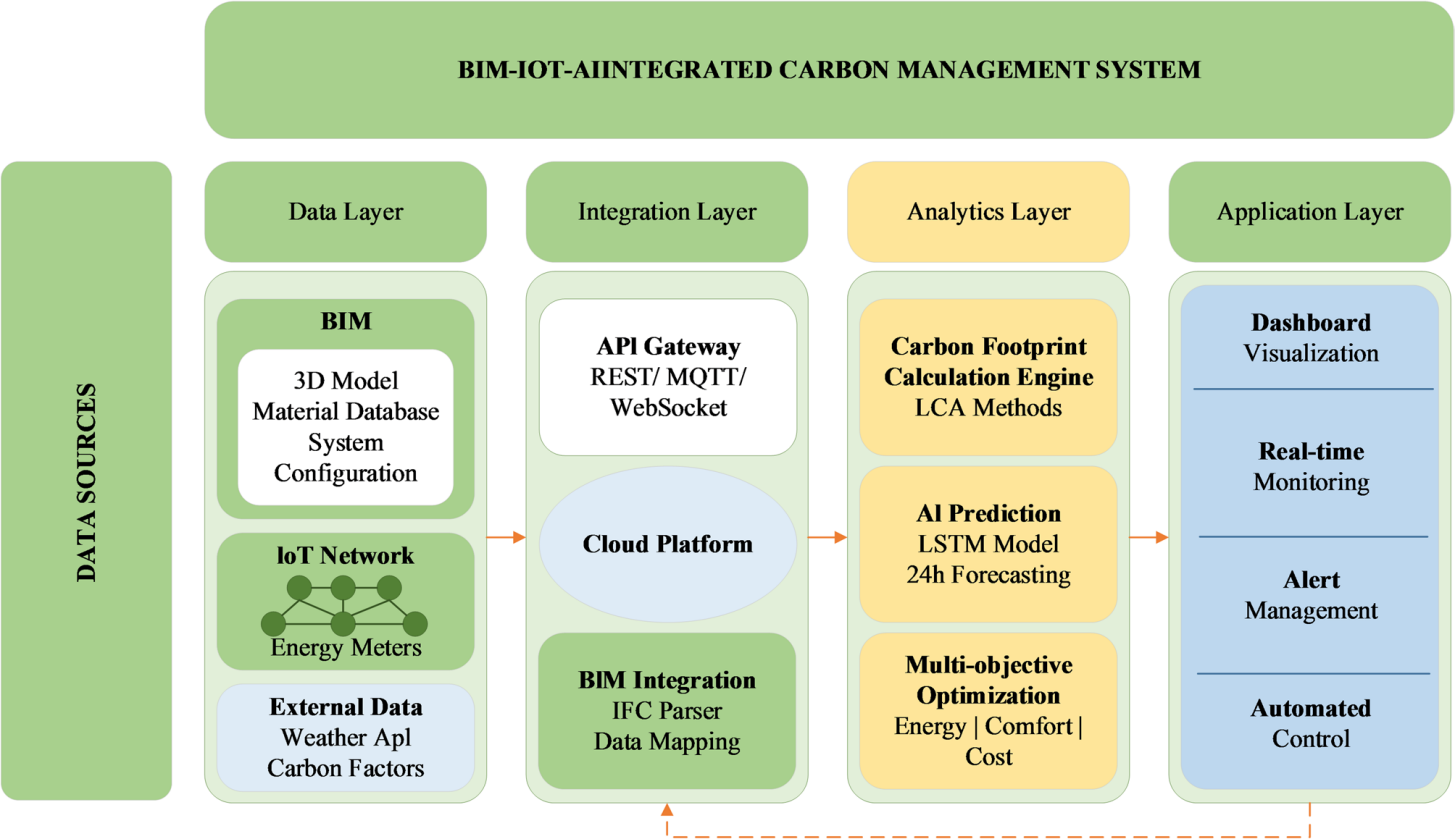
BIM-IoT-AI integrated system architecture for real-time carbon footprint assessment.

The system design introduced uses a four-layer system that has been designed to achieve interoperable integration of Building Information Modeling (BIM), Internet of Things (IoT) sensors, as well as artificial intelligence (AI) algorithms, for real-time carbon footprint evaluation purposes. As indicated in Fig. 2, the system has the data layer, integration layer, analytics layer, as well as application layer, which play particular functions while ensuring that there exists continuous data flow throughout the system.

In essence, the data layer aggregates data from three primary sources. Rich static building informations such as 3D models, material properties, as well as system configurations, are retained in the BIM databases, while IoT sensor networks provide real-time readings from energy meters, environmental sensors, as well as occupancy sensors. Contextual data such as weather predictions as well as carbon emission factors are provided through external data sources. The multi-source methodology, as an extension of some of the recent literature on integration of BIM-IoT^17^, addresses the requirement of both static as well as dynamic data requirements of carbon analysis. The system integration layer is its middleware that presents API gateway presence through various communication protocols such as REST for web services, MQTT for lightweight IoT communications, as well as WebSocket for bidirectional real-time data transfer^18^. Scalable data processing, as well as data storage, are provided through the cloud platform of the layer, while specialized integration modules of BIM with IFC parsing facilities make provision for standardized interpretation of data.

The analytics layer functions as the central component for data processing and integration, transforming heterogeneous raw data into structured and actionable information. Standardized LCA procedure is used on the carbon-footprint-calculated module for both embodied & operational carbon calculation A 24 h forecast was provided by the AI forecasting module with LSTM neural networks, and the multiobjective optimization controls at the same time, the energy efficiency, occupant comfort and operation expenditures^19^. These components operate collaboratively to enable proactive carbon management, rather than relying on reactive monitoring. At the application layer, it provides users with visualization dashboards, real-time monitoring boards, and automatic control functions. In this feedback optimization loop, the system keeps learning as well as continuously optimizing carbon control.

### BIM-IoT integration module

**Fig. 3:**
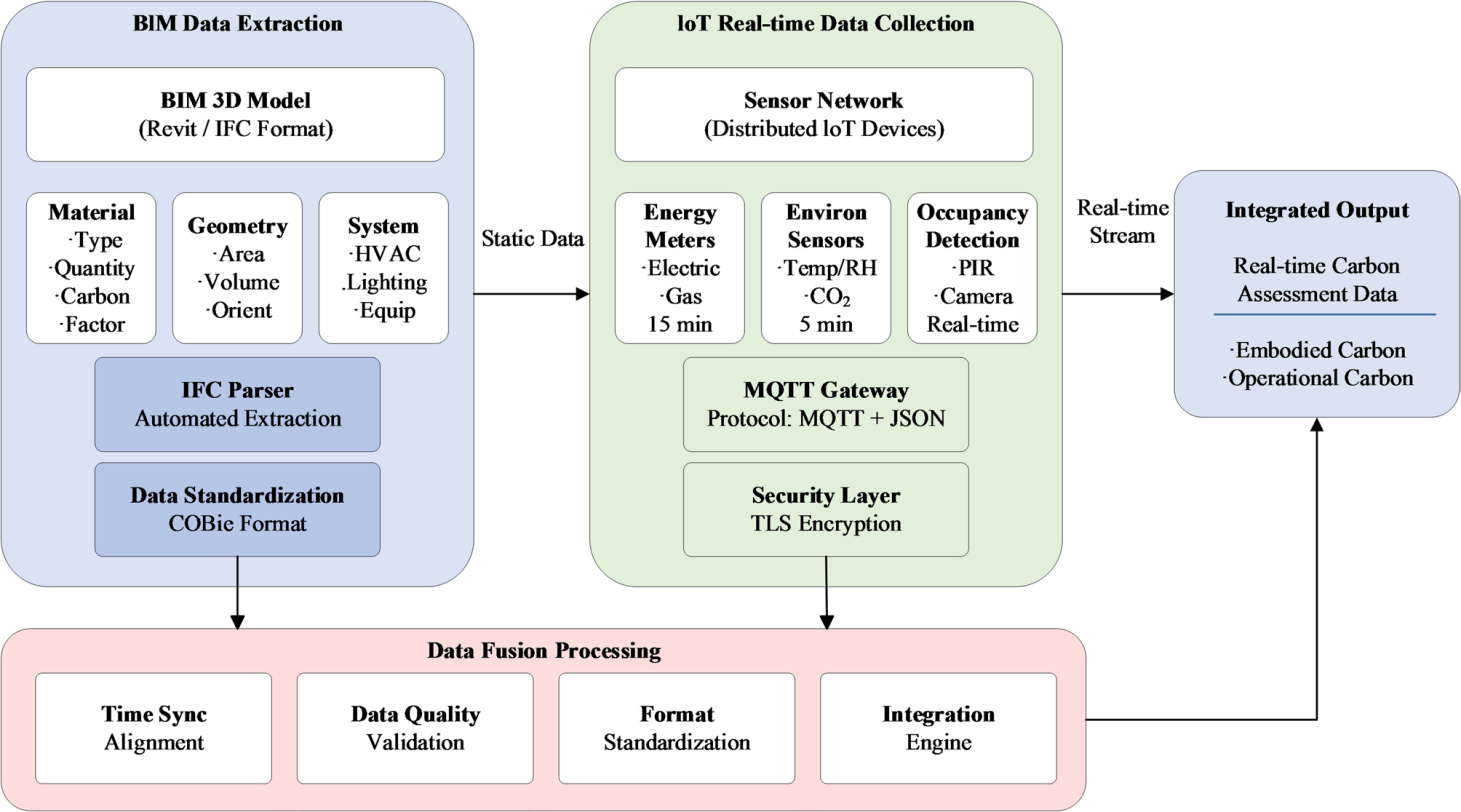
BIM-IoT data integration flow diagram.

BIM-IoT integration module is the foundation for obtaining a full set of data. It realizes the interaction between static building information and dynamic building status. As can be seen from Fig. 3, the module is made up of 2 major elements that are coexisting to provide us with real time Carbon Footprint Evaluation feature.

BIM data export module uses Industry Foundation Classes (IFC) formats to export primary building data automatically from 3D models. The material properties such as material type, quantity, as well as embodied carbon values, are acquired through automated algorithms, which form primary data used in embodied carbon calculation^20^. Geometric properties such as floor area, volume, as well as building orientation, are acquired at the same time to facilitate accurate energy modeling as well as environmental analysis. System configurations such as HVAC details, lighting schedules, as well as equipment, are acquired for setting baseline operation parameters^21^.

IoT’s real-time monitor consists of a distributed sensor network within the building that detects the time varying operation. Energy Meter records both electricity and NG on a fifteen-minute basis so there is very high-gridded energy consumption The environmental sensors read temperature, humidity and CO2 every 5 min and enable environmental quality in- doors (as a proxy) vs. energy consumption correlations^22^. The sensor network uses MQTT with JSON data formats for a small lightweight, but still interoperable between different IoT device data transport. The TLS encryption gives secure information & it gives integrity of data during transferring & removes big security problems in cloud designs^23^.

It does the matching of BIM-static information and IoT-dynamic flows through a temporary matching process and data verifying methods And harmonized to enable real time calculation of the carbon footprints of embodied carbon of materials and operation carbon of energy use for comprehensive evaluation frameworks more than current static evaluation. All sensor configurations, data acquisition frequencies, communication protocols, and data preprocessing procedures are standardized and documented. All sensor configurations, data acquisition frequencies, communication protocols, and data preprocessing procedures are explicitly specified and standardized, ensuring that the BIM–IoT integration process is fully reproducible and can be independently validated in other buildings with comparable sensor infrastructures.

### Carbon assessment model

The carbon assessment model adopts a dual-track approach to account for both embodied and operational carbon emissions over the building life cycle. By integrating BIM-derived material information with real-time operational data collected through IoT sensors, the model enables a comprehensive representation of carbon emissions associated with both construction and use phases.

Embodied carbon calculation refers to the comprehensive assessment of emissions associated with material production, transportation, and installation. The model uses the equation:

1 $$EC = \sum\limits_{i = 1}^{n} {(M_{i} \times EF_{i} \times (1 + W_{i} ) \times T_{i} )}$$

where $M_{i}$ represents the quantity of material $i$, $EF_{i}$ denotes the emission factor for material production, $W_{i}$ accounts for material waste coefficients, and $T_{i}$ incorporates transportation-related emissions. It is based on traditional LCA formulations but uses material amounts of BIM to be used alongside regional-specific EF for more accurate evaluation of embodied carbon^24^. Particular attention is given to waste-related inefficiencies, which are often overlooked in conventional calculations, so using them makes your final picture match the real-world way materials go into place for prefab structures.

Operational carbon assessment using real-time IOT data flow to assess change in buildings operations. The model employs:

2 $$OC = \sum\limits_{j = 1}^{m} {(E_{j} \times CF_{j} \times t)}$$

where $E_{j}$ represents energy consumption from source $j$, $CF_{j}$ indicates the carbon factor for the specific energy type, and $t$ denotes the time interval. This method can consider the change trend of the energy consumption pattern over time. The carbon intensity of the grid will vary at any time during the day^25^. IoT sensor data are integrated to enable continuous monitoring and rapid identification of carbon emission hotspots. This enables proactive optimization of building operations.

The total life-cycle carbon footprint is obtained through aggregation of embodied and operational emissions, with adjustments applied to ensure temporal and system boundary consistency. The capability to process both static BIM-derived data and dynamic IoT data streams enables the assessment framework to reflect actual building performance more accurately than static LCA approaches. As such, the model is intended as a practical assessment tool for operational carbon management rather than as a redefinition of established LCA methodologies^26^.

### Optimization framework

The optimization framework functions as a decision-support layer that translates real-time carbon assessment outputs into operational adjustment strategies. Rather than acting as an autonomous control entity, the framework is designed to support predictive carbon management by coordinating operational decisions under multiple, and often competing, constraints. These include minimizing operational carbon emissions while maintaining occupant comfort and acceptable energy efficiency. The emphasis is placed on feasibility and responsiveness in real-world building operation, rather than on algorithmic optimality or autonomous control.

Carbon emission forecasting within the framework is supported by a Long Short-Term Memory (LSTM) neural network, selected for its suitability in modeling temporal dependencies in building operation data at fine temporal resolutions. Prior studies have demonstrated that LSTM-based architectures can achieve prediction accuracies exceeding 90% for 24-h industrial or building-scale carbon emission forecasting tasks^27^. In this study, the predictive model learns operational patterns from historical energy consumption data while adapting to seasonal variations and occupancy schedules. More advanced architectures, such as temporal convolutional networks (TCNs) or attention-based models, have been reported to yield improved performance in certain peak-demand scenarios due to enhanced feature selection capabilities^28^. However, such models typically involve increased computational complexity, which may limit their practical deployment in continuous, real-time operational settings.

The optimization strategy adopts a multi-objective formulation to explicitly balance competing operational objectives, including carbon emission intensity, energy cost, and thermal comfort. A weighted objective function is employed and updated dynamically to reflect changes in operational conditions and grid carbon intensity. This formulation was selected to accommodate the inherently conflicting nature of building performance objectives in occupied environments, where single-objective energy minimization approaches are often insufficient. By contrast, the adopted multi-objective framework enables adaptive trade-offs that are better aligned with real-time operational decision-making requirements^29^.

The forecasting module is integrated into the optimization process to support predictive, rather than purely reactive, operational control. By anticipating short-term emission trajectories, the framework enables pre-emptive adjustments to building operation before inefficiencies materialize. Previous studies have indicated that foresight-based control strategies can yield measurable improvements over reactive approaches when reliable short-term predictions are available^30^. In this context, the predictive optimization capability is intended to enhance operational robustness and responsiveness, rather than to provide formal guarantees of optimal or globally minimal emission outcomes.

### Case study setup

The case study conducted on a LEED gold-rated office building to prove the proposed bim-IoT hybrid carbon assessment framework. Building, as considered, is a single ordinary commercial building of the sub-tropical climatic zone, having all the data available for a system assessment, as well as calculations.

The way it was carried out was in stages, starting off by shaping a through and thorough BIM model which has all the materials specified, geometric attributes, and the systems all laid out. Over the past implementations of sustainable BIM technology in construction projects has shown that Digital Models that are comprehensive are the base of effective Carbon Management Systems^31^. The BIM model was checked against asbuilt drawings and modified to reflect actual building materials and quantities.

The deployment of IoT sensor network comprised in total 150 devices spread around a building collecting info about energy consumption as well as environmental and occupancy data. Smart meters were installed at the primary electrical panels and sub-metering locations. Monitoring of energy use was recorded every 15 min. Environmental sensors for temperature, humidity, and CO_2_ concentrations were put in all living spaces and collected data every 5 min. In the deployment strategy it took some successes from Indonesia’s cases which emphasized the necessity of enough sensor coverage to get valid sustainability evaluation^32^.

Table 1 lists the key features of the case study building and the implementation parameters. As can be seen from Table 1, the monitoring time is selected for twelve months to cover the seasonal factors. Establish the comprehensive baseline data. The big database of more than 1 million data points was used to check on carbon assessment formulas and on ways to make the assessments better. Data quality protocols were in place to guarantee more than 98% accurate measurements which became good inputs for calibrating and testing the model. To support verification and reproducibility, detailed descriptions of sensor deployment, data collection intervals, data preprocessing procedures, and model training configurations are provided, enabling the proposed framework to be replicated and validated in other real-world building contexts with comparable data availability.

**Table 1:** Case study building characteristics and implementation parameters.

Parameter	Value
Building type	Commercial office
Total floor area	15,000 m^2^
Number of Floors	12
LEED certification	Gold
Climate zone	Subtropical (Cfa)
Monitoring period	12 months
Total sensors	150
Energy meters	15
Environmental sensors	45
Occupancy detectors	30
Data collection points	> 1,000,000
Data accuracy	> 98%

## Results

### System performance validation

**Fig. 4:**
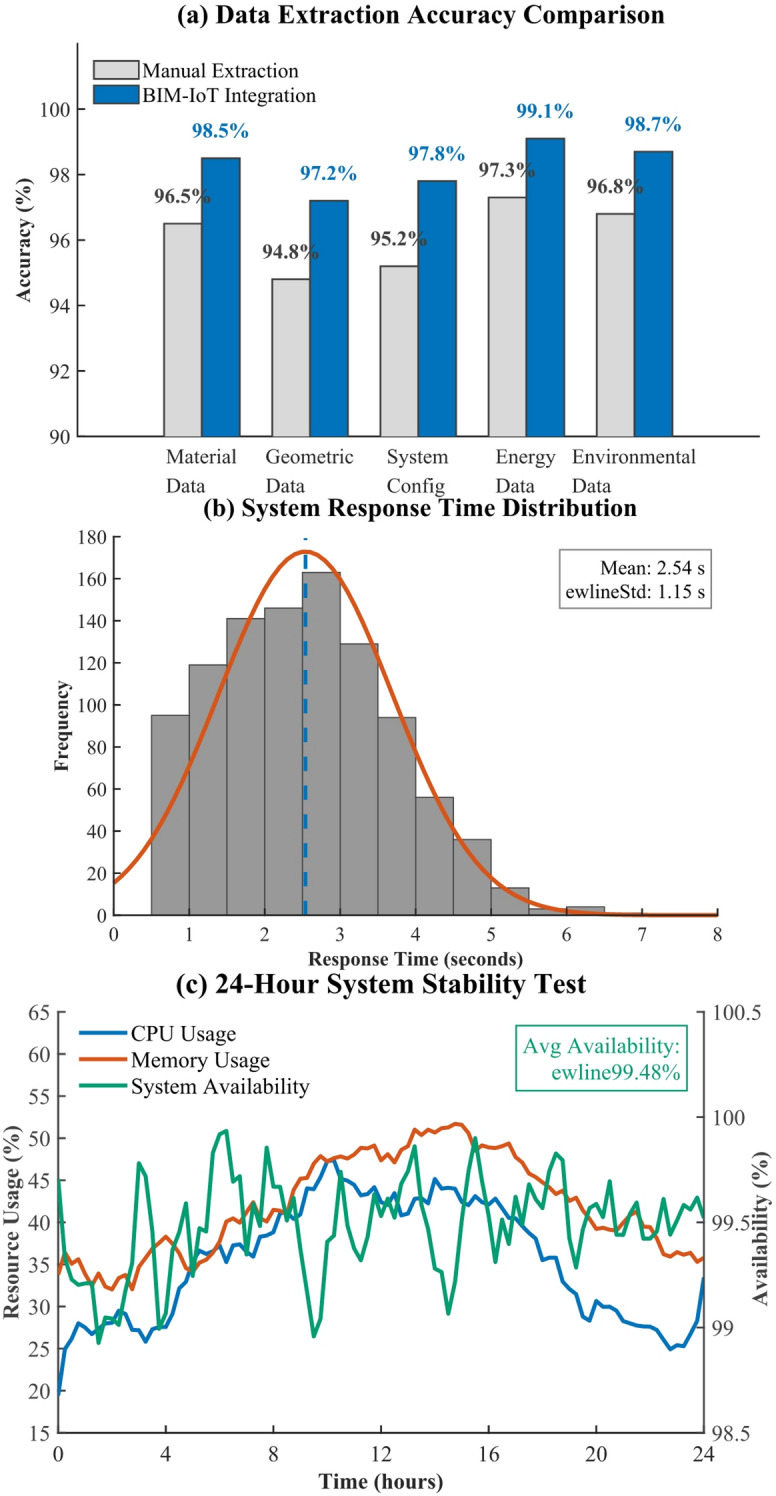
System performance validation. (**a**) data extraction accuracy comparison, (**b**) system response time distribution, and (**c**) 24-h system stability test.

The system performance validation proves the system’s strong capability of performance. Testing is done through an extensive number of performances metrics. As shown in Fig. 4, the validation mainly contains extraction accuracy of the system data, characteristics of response time of the system and long term stability of the system operation.

Data extraction accuracy comparisons indicate that improvements via automated BIM-IoT integration are far superior to the manual approaches. The integrated system constantly achieves an advantage over manual extraction, and the higher the data category, the greater the improvement is, the material data accuracy has improved from 96.5 to 98.5%, geometric data has improved from 94.8 to 97.2% and system configuration data has improved from 95.2 to 97.8%. Energy and environmental data is extracted, and the extraction result is very high, reaching an extraction precision of 99.1%, and an extraction precision of 98.7% respectively, such improvements make known that automation of parsing algorithms and data protocol standards reduces human errors and maintains data in good condition.

System response time analysis indicates that the proposed framework meets the requirements for real-time operational monitoring. Response times exhibit an approximately normal distribution with a mean of 2.54 s and a standard deviation of 1.15 s. The majority of responses fall within the 2–4 s range, which is substantially shorter than the 15-min data acquisition interval used for energy monitoring. This temporal margin ensures that incoming IoT data streams can be processed without backlog or latency accumulation under the examined operating conditions.

A 24-h stability test further confirms the reliability of the system under steady-state operation. During the test period, CPU utilization ranged between 25 and 50%, indicating adequate computational headroom relative to peak load requirements. Memory utilization remained stable at approximately 40–45%, suggesting efficient resource management without evidence of memory leakage. System availability averaged 99.48% over the test duration. Minor availability fluctuations were primarily associated with scheduled data synchronization and backup processes, which did not interrupt real-time monitoring functions.

Taken together, these validation results indicate that the integrated BIM–IoT framework provides a stable and responsive platform for continuous carbon footprint assessment. The observed performance characteristics support its suitability for long-term operation with consistent data quality and timely processing, rather than implying superiority beyond the tested deployment context.

### Carbon footprint assessment

**Fig. 5:**
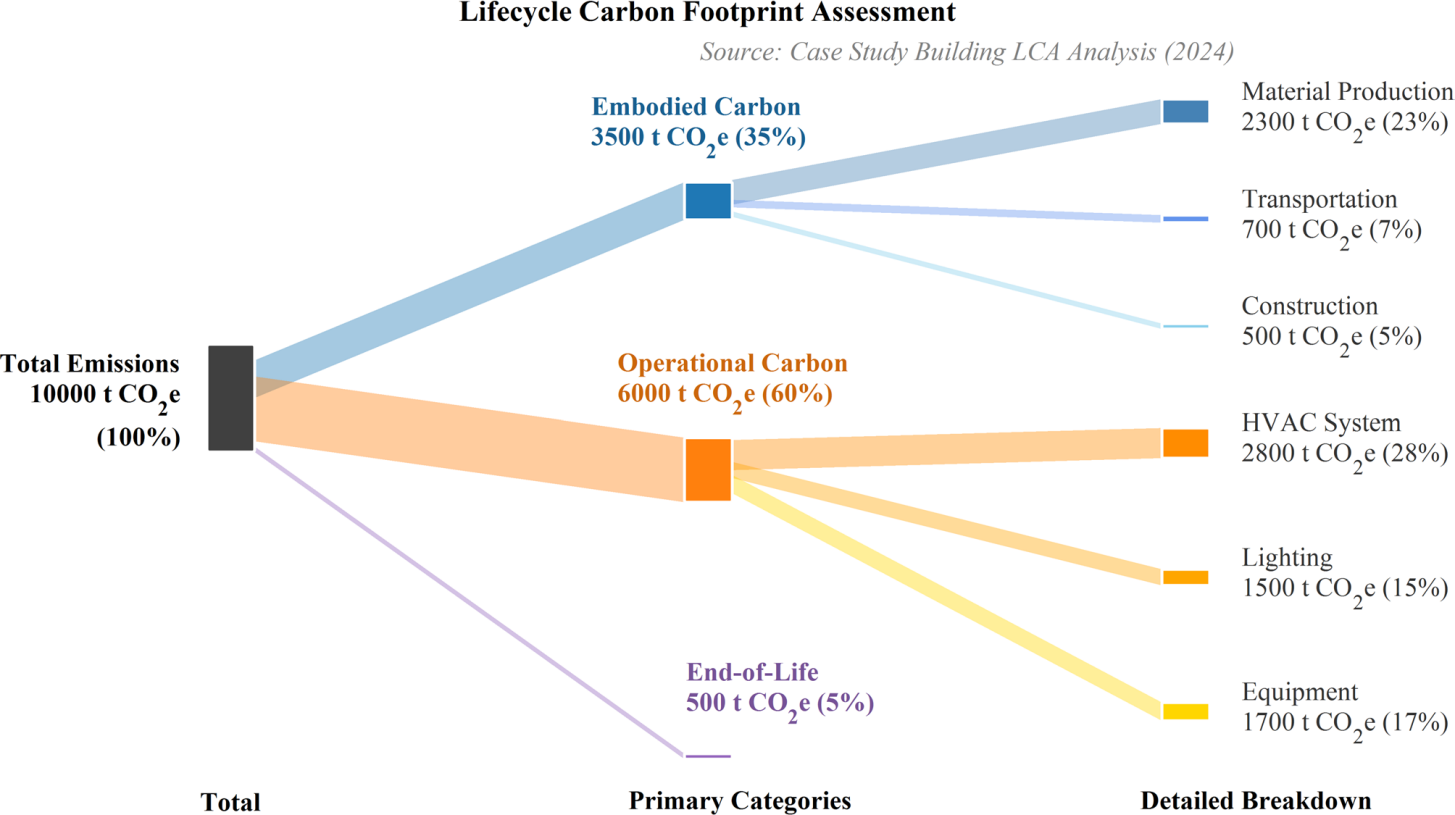
Lifecycle carbon footprint distribution of the case study building.

Whole-of-building life cycle carbon footprint of case building exhibits clear emission curves through various phases as well as various building elements, which exhibit significant carbon mitigation potentials as shown in Fig. 5. The total carbon footprint is distributed across embodied, operational, and end-of-life stages, with operational emissions representing the dominant share. This distribution aligns with global building sector trends while highlighting the dominant role of operational emissions in the overall carbon profile.

As illustrated in Fig. 5, embodied carbon is predominantly driven by material production, while transportation and construction contribute comparatively smaller shares, highlighting the importance of material-related mitigation strategies. The BIM-linked assessment method mad to make exact counting with automatic material take-offs and region -specific emission -factor apps.

For operational carbon, the largest is HVAC at 28% (2800 tCO_2_e) then comes the equipment and plug loads at just shy of a 17% (1700 tCo2e) and Lighting systems coming in at just below 15% (1500 tCo2e). In terms of the real-time monitoring capabilities of the IoT Sensor Network, major variations throughout time can be seen in these emissions with highest consumption taking place when in times of a building occupancies schedule and external climates. From Table 2, one can be noted that the monthly carbon emissions shows seasonal pattern, the minimum monthly carbon emissions is 45.2t CO2e in April whereas the maximum is 68.7t CO2e in August. Large carbon emissions are mostly because of the cooling demand caused by the subtropical climate.

**Table 2:** Monthly carbon emissions breakdown by category (tCO_2_e).

Month	HVAC	Lighting	Equipment	Total operational	Monthly total
January	22.3	11.8	14.2	48.3	51.5
February	20.1	10.9	13.5	44.5	47.6
March	21.4	11.2	13.8	46.4	49.5
April	19.8	10.5	12.9	43.2	45.2
May	24.6	11.3	14.1	50.0	53.1
June	28.3	12.1	15.2	55.6	58.7
July	31.2	12.8	16.3	60.3	63.4
August	32.5	13.2	16.8	62.5	68.7
September	27.9	12.4	15.5	55.8	58.9
October	23.7	11.6	14.3	49.6	52.7
November	21.5	11.1	13.7	46.3	49.4
December	22.8	11.9	14.5	49.2	52.3

Using the dynamic assessment approach has showed the emission patterns that the static assessment approaches would not usually detect. Weekend and after-hours energy consumption made up around 18% of the total operational emissions, so there is quite a bit of potential for improvement when it comes to scheduling and controlling these times. Moreover, integrating real-time grid carbon intensity data shows that identical energy consumption can have emission rates up to 15% difference on different times of the day and different renewable energy sources in the grid.

### Real-time vs static comparison

**Fig. 6:**
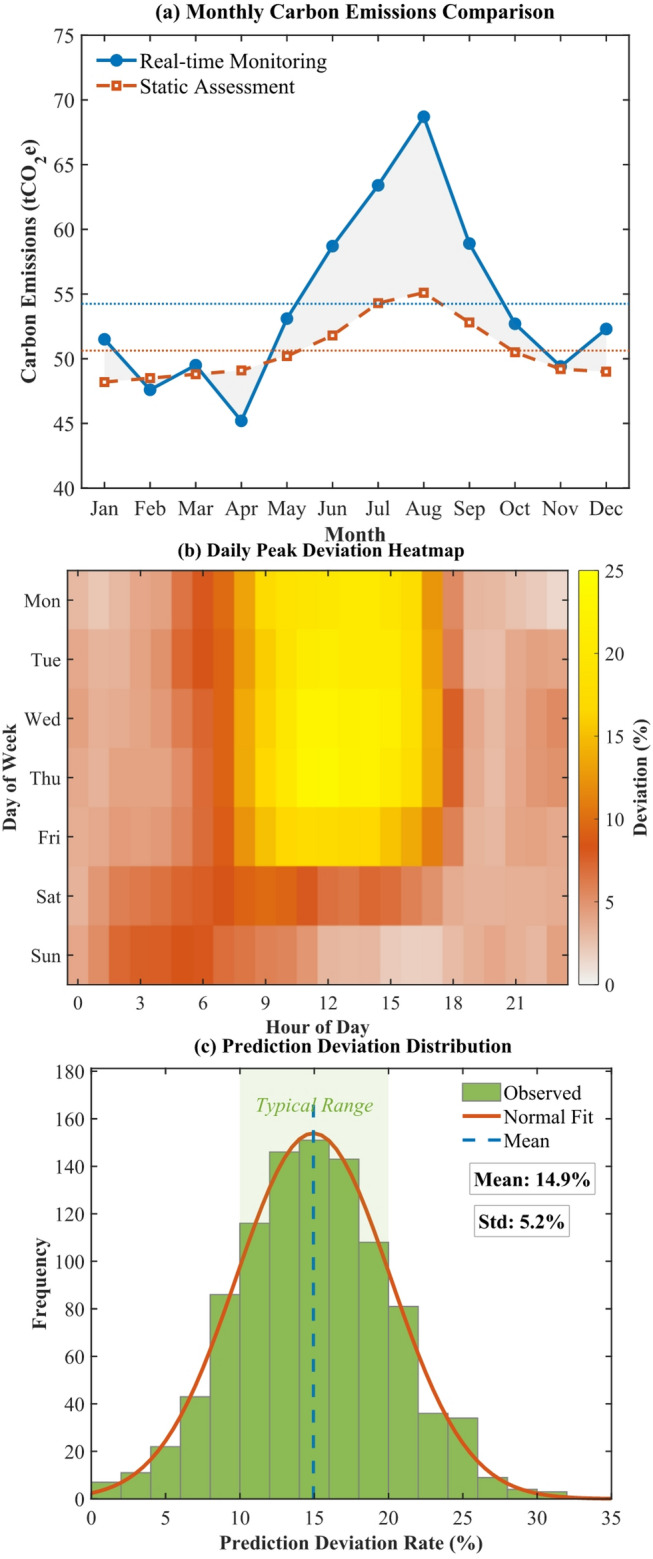
Comparison between real-time monitoring and static assessment approaches.

Real-time Monitoring vs Static Assessments: Significant differences are observed in carbon emission quantification when comparing real-time monitoring with static assessment approaches. The latter proves to have shortcomings. As we can see from above, Fig. 6 shows that the Dynamic Monitoring System is able to catch and observe the temporal and operational complexities which Static Analysis has a systematic tendency to under-estimate or even ignore.

The monthly carbon emissions comparison (Fig. 6a) shows strong seasonal variations in the real time emissions, ranging from a low of 45.2 tCO_2_e in April to a high of 68.7 tCO_2_e in August. This is a large change of 52% between lowest and highest emission. As opposed to these being static, they maintain almost consistently regular predictions in the range 50–55tCO2e, yearly. Not considering climate related changes on their energy demand: The situation shows more distinct differences when it is summer, real time monitoring records show max emissions are 23–25% greater than static predations, mosty because of the need to cool more because of the subtropical weather. Similar to what was discussed with regard to transitions, under-estimates happen due to the fact that static models use a regular operations paradigm irrespective of whether an external “environment” conditions are met.

Daily Peak Deviation Heatmap (Fig. 6b), this can reveal critical temporal patterns in how accurate the predictions are at various times of the day. Weekday office hours (9:00–18:00) show the largest deviation rates, 20–25% which shows that stationary models are unable to represent the difference caused by occupancy in energy use. Weekend shows a drop yet still a big departure from 5 to 10% which points out that the base building loads plus the uses which occur in a fitful manner aren’t well-represented inside static evaluations. The striking contrast between peak hour and off-peak hour shows that time resolution is very important in carbon footprint calculation because static model used an average emission factor, neglecting those important differences.

Statistical analysis of prediction deviation rate (Fig. 6c), we can see that it is a normal distribution with a deviation mean of 14.9% and a deviation standard of 5.2%, showing that the static evaluation method has under-estimated the result overall. From the distribution, around 68%of the observations can be seen falling between–the 10–20%deviation range, with extreme deviations of more than 25% happening in only about 5% of cases, this being mostly during unusual operational events or extremely weather conditions. It goes along with older study that saw static LCA ways of figuring things out might undercount the operation carbon emissions by 15–20% due in large part to simpler ideas of how a building actually works.

This discrepancy highlights important implications for practical environmental management and operational decision-making. Real-time monitoring allows previously unknown emissions such as parasitic loads when unoccupied or efficiency drops at partial load conditions. Also, the dynamic assessment strategy helps to carry out flexible optimization plans to reach a reduction rate between 10 and 15% of emission sources through the process of rectification when there is high deviation period.

### Optimization outcomes

**Fig. 7:**
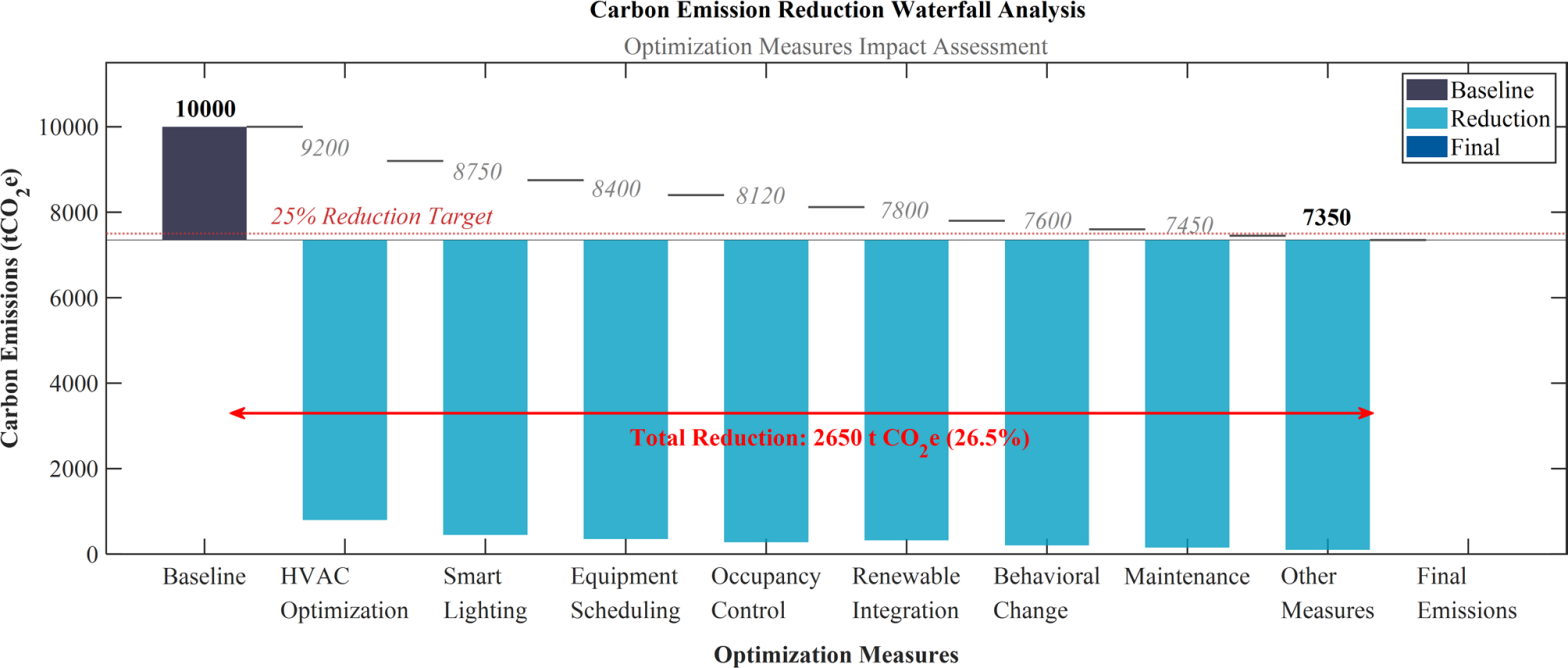
Carbon emission reduction waterfall analysis showing cumulative optimization impact.

Following the deployment of the integrated BIM–IoT–AI framework, measurable reductions in operational carbon emissions were observed in the studied building. As illustrated in Fig. 7, the waterfall analysis indicates a reduction from an operational baseline of 10,000 tCO_2_e to approximately 7,350 tCO_2_e over the assessment period, corresponding to a case-specific reduction of 26.5%. This reduction reflects the cumulative effect of multiple coordinated operational adjustments rather than the impact of any single optimization measure. Among the identified contributors, HVAC operational optimization accounted for the largest share (approximately 800 tCO_2_e, 8%), followed by smart lighting control strategies (approximately 450 tCO_2_e, 4.5%) and equipment scheduling adjustments (approximately 350 tCO_2_e, 3.5%). These results should be interpreted as indicative of the relative contribution of different operational domains within the specific building context examined.

Optimization benefits’ time course shows progressively greater benefits with more data in system’s operation and improvements to its predictive algorithms. In the short term, there will be an improvement of 10–15% through immediate changes to operational parameters and eliminating obviously bad practices. The medium time frame with monthly optimization cycles yields an increase in 15–20% as the system learns more about how we use the buildings and also learn the seasonal changes. For a long term, it can achieve an annual yield of 20–25%, which is close to the upper limit without sacrificing the comfort of the occupants.

The economic analysis proves there is strong financial support for the implementation of the system, from Table 3. The 1.10 million yuan for the opening capital investment includes the expenses for hardware installation, software license, and system integration. The annual operations saving of 0.65 million comes from the less energy consumed, better scheduled maintenances and less carbon tax burden. The payback period that results is just 1.7 years, far better than the average building retrofit investments often need three to five years for them to pay for themselves.

**Table 3:** Economic analysis of optimization implementation.

Parameter	Value	Unit	Remarks
Initial investment	1.10	Million Yuan	Hardware, software, integration
Annual energy savings	0.52	Million Yuan	Based on current tariffs
Maintenance savings	0.08	Million Yuan	Predictive maintenance benefits
Carbon credit revenue	0.05	Million Yuan	At 50 Yuan/tCO_2_e
Total annual savings	0.65	Million Yuan	Combined benefits
Simple payback period	1.7	Years	Without discount rate
Net present value (10 years)	4.25	Million Yuan	At 5% discount rate
Internal rate of return	42	%	10-year evaluation period

The observed effectiveness can be attributed to the framework’s ability to identify and exploit previously unrecognized emission reduction opportunities. Real-time monitoring indicates that behavioral adjustments and renewable energy integration contribute modest but measurable emission reductions. However, when these two measures combine with technical ones they produce a synergetic effect. Additionally due to systems ability to predict it can actively shift it’s load to periods which have lower grid carbon intensity levels which is lower emissions than what would be capture in single static assessment. It should be noted that the observed emission reductions are not attributed to a single isolated intervention. Rather, they result from a set of operational measures systematically identified, coordinated, and optimized through the proposed BIM–IoT–AI framework, which functions as an enabling platform for informed decision-making rather than a standalone emission reduction technology.

### Prediction model performance

**Fig. 8:**
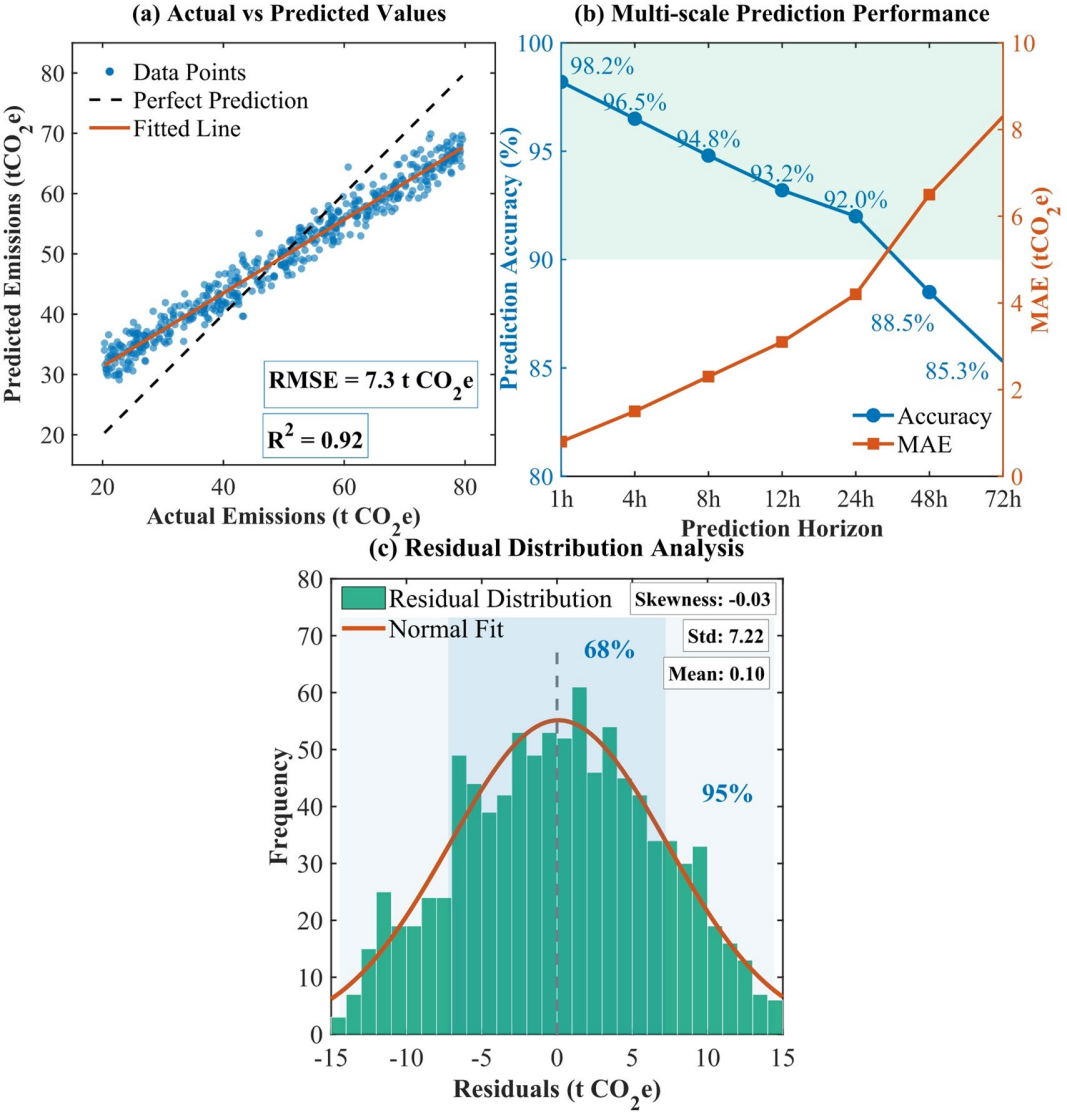
LSTM prediction model performance evaluation.

The LSTM-based prediction model shows great performance in predicting the carbon emissions of buildings at various time scales, the model is effective for real time optimization applications. From Fig. 8‘s all the comprehensive evaluation metrics can verify that the model is able to capture the complicated temporal patterns without compromising accuracy levels at an operational decision-making.

Scatter plot analysis (Fig. 8a) shows a strong agreement between predicted and observed carbon emissions, with an R^2^ value of 0.92 and an RMSE of 7.3 tCO_2_e. The fitted regression line closely follows the ideal 1:1 reference line, suggesting the absence of pronounced systematic overestimation or underestimation. This result indicates that approximately 92% of the variance in observed emissions can be explained by the model under the studied conditions. The observed predictive performance is attributable to the model’s ability to learn non-linear relationships among integrated inputs, including occupancy-related operational patterns, short-term weather variations, and historical energy consumption profiles, rather than to any algorithmic novelty.

Multi-scale prediction performance evaluation (Fig. 8b) shows the expected gradual loss in accuracy over longer forecasting periods. Short-term predictions keep great accuracy at 98.2% for 1-h forecasts, decreasing slowly to 85.3% for 72-h forecasts. So the MAE, or mean absolute error, which is another measure of prediction accuracy, jumps up from 0.8 tCO2e all the way to 8.3 tCO2e over the very same time range. The intersection point, when accuracy dips below 90%, which is at 36 h, that’s the point of practicality for a workable operational plan. It lines up with the buildings’ capacity to endure heat and how we can understand people coming and going for at least two days at a time.

Residual Distribution Analysis (Fig. 8c) shows near a perfect normal distribution of residuals to prove statistical correctness of model predictions. The residuals have an average number of 0.10 tCO_2_e, nearly nothing with respect to measurements, as well as a standard deviation of 7.22 tCO_2_e. The near zero skewness -0.03 means that there is no systematic tendency for the error to be positive or negative. The error is symmetrically distributed. From Table 4, around 68% of predictions are between ± 1σ(± 7.22tCO2e) and around 95% are between ± 2σ(± 14.44tCO2e), which is in line with the theoretical normality requirements, meaning the model is statistically reliable.

**Table 4:** LSTM model performance metrics across validation datasets.

Metric	Training set	Validation set	Test set	Units
R^2^ score	0.94	0.92	0.92	–
RMSE	6.8	7.3	7.3	t CO_2_e
MAE	5.2	5.6	5.7	t CO_2_e
MAPE	8.4	9.1	9.2	%
24 h accuracy	93.5	92.0	92.0	%
Inference time	–	–	0.12	seconds

The model performs effectively due to its ability to capture both short-term dynamics and long-term seasonal patterns through the LSTM architecture. A network’s memory cells hold onto the details of what is going on in the heat things for buildings to do about it all—including stuff related to things like a day where some part was warming up at sunrise or maybe a night where they took it easy as it went to sleep. And make these abilities place the predicting model on the essential piece for ahead of schedule carbon operation plans.

Despite the strong predictive performance observed under normal operating conditions, it should be noted that the LSTM model may exhibit limitations when handling anomalous events, such as extreme weather conditions or unexpected operational disruptions. These events are typically underrepresented in the training data, which may reduce prediction accuracy and increase uncertainty during such periods. This limitation is consistent with the observed decline in prediction accuracy over longer forecasting horizons and highlights the need for caution when applying the model under non-typical operating scenarios.

## Discussion

Applying the proposed BIM–IoT–AI integrated framework reveals operational carbon emission characteristics that are typically not captured by conventional static assessment approaches. In the studied building, an operational emission reduction of 26.5% was observed under the specific climatic, occupancy, and control conditions considered. This reduction exceeds the 15–20% range commonly reported for conventional retrofit measures, suggesting that continuous monitoring and operational optimization can uncover additional, case-specific mitigation opportunities^33^. Furthermore, the identification of approximately 18% of operational emissions occurring during non-working hours highlights the presence of persistent base loads and parasitic energy consumption, reinforcing the value of continuous IoT-based monitoring for operational diagnostics rather than serving as evidence of universal performance gains^34^. The results indicate that the framework is particularly effective in detecting and responding to short-duration operational inefficiencies that would likely remain undetected under coarse temporal resolution assessment methods.

The forecasting performance achieved in this case study further supports the operational role of predictive analytics within the framework. A 24-h prediction accuracy of approximately 92% was obtained, which is higher than the 80–85% accuracy range commonly reported in comparable building energy forecasting studies^35^. This performance can be attributed to the ability of the LSTM architecture to capture both short-term operational dynamics and longer-term seasonal patterns when trained on integrated BIM-derived contextual information and IoT time-series data^17^. Nevertheless, prediction accuracy decreased to approximately 85.3% for a 72-h horizon, reflecting the inherent uncertainty associated with human behavior and weather variability, as widely acknowledged in machine learning–based building energy studies^36^. These results indicate that the forecasting module is suitable for short-term operational decision support, rather than long-range deterministic prediction.

When compared with optimization strategies focusing on isolated subsystems, such as envelope-level interventions, the integrated operational approach demonstrates a comparatively higher reduction potential within the specific case examined. For example, Kamazani and Dixit reported a 22% carbon reduction through envelope optimization alone^37^, whereas the present study observed additional reductions through coordinated operational adjustments across multiple systems. From an economic perspective, the observed simple payback period of approximately 1.7 years is shorter than the 3–5 year range typically associated with capital-intensive physical retrofits, indicating that operational optimization can offer a cost-effective complement to traditional retrofit strategies rather than a direct replacement^38^. Conceptually, the framework extends static LCA-based assessment toward a continuous, operation-oriented carbon management approach, while addressing several BIM–IoT integration challenges identified in prior studies^10,16^.

Despite these promising results, several limitations must be acknowledged. The initial investment cost, estimated at approximately 1.10 million Yuan in this case, may present a barrier for smaller buildings, consistent with challenges reported for IoT-enabled energy management systems in the literature^34^. In addition, data privacy and cybersecurity requirements introduce non-trivial implementation complexity that must be addressed through appropriate technical and organizational safeguards^19^. The lack of fully standardized BIM–IoT data exchange protocols also limits seamless scalability across building portfolios, often necessitating project-specific integration efforts^32^. Moreover, the robustness of the proposed forecasting and optimization framework under extreme or anomalous weather conditions has not yet been validated and warrants further investigation^30^.

Future research will focus on extending the framework toward district-scale applications, where coordinated optimization across multiple buildings may support load balancing and shared emission reduction benefits. Transfer learning techniques could reduce data requirements when deploying the framework in new buildings, thereby lowering implementation barriers^27^. Additional exploration of technologies such as blockchain-based carbon credit verification and federated learning may further enhance transparency and privacy preservation. As BIM–IoT data exchange standards mature, as highlighted in recent reviews on sustainable construction technologies^15^, the practical applicability and interoperability of real-time carbon management frameworks are expected to improve. Overall, the present findings should be interpreted as case-based evidence of feasibility and potential, rather than as generalized performance claims.

These results provide empirical validation of the proposed framework under real-world operational conditions, supporting its practical applicability rather than relying solely on theoretical or simulation-based evaluation.

## Conclusions

This study developed and validated an integrated BIM–IoT–AI framework for real-time carbon footprint assessment and operational optimization in sustainable buildings. In a real-world case study, the framework reduced total carbon emissions by 26.5% relative to a 10,000 tCO_2_e baseline, surpassing the targeted 20–25% reduction while maintaining acceptable occupant comfort. The LSTM-based prediction module achieved 92% accuracy for 24-h forecasts, and continuous monitoring showed that roughly 18% of operational emissions occurred during unoccupied periods—highlighting the value of dynamic monitoring compared with conventional static assessment. An economic evaluation further supported practical feasibility, with a simple payback period of 1.7 years and an internal rate of return of approximately 42%, outperforming typical building retrofit investments.

Beyond the outcomes of a single building, the proposed framework offers operators actionable, data-driven capabilities for real-time carbon management. By enabling timely detection of operational inefficiencies and supporting informed control decisions, the framework facilitates more responsive and effective emissions-reduction strategies. The observed link between continuous monitoring and improved emissions performance also provides practical guidance for enhancing operational practice and informing performance-oriented carbon management policies. Overall, the results underscore the potential of digital, real-time carbon management to accelerate decarbonization in the building sector.

## Data Availability

The datasets generated and analyzed during the current study are not publicly available due to data confidentiality agreements with the building owner, but are available from the corresponding author upon reasonable request.
